# microRNA regulation of the embryonic hypoxic response in *Caenorhabditis elegans*

**DOI:** 10.1038/srep11284

**Published:** 2015-06-11

**Authors:** Konstantinos Kagias, Roger Pocock

**Affiliations:** 1 Biotech Research and Innovation Centre (BRIC), University of Copenhagen, Ole Maaløes Vej 5, Copenhagen, Denmark; 2 Department of Anatomy and Developmental Biology, Faculty of Biomedical and Psychological Sciences, Monash University, Clayton, Victoria, Australia

## Abstract

Layered strategies to combat hypoxia provide flexibility in dynamic oxygen environments. Here we show that multiple miRNAs are required for hypoxic survival responses during *C. elegans* embryogenesis. Certain miRNAs promote while others antagonize the hypoxic survival response. We found that expression of the *mir-35* family is regulated by hypoxia in a HIF-1-independent manner and loss of *mir-35–41* weakens hypoxic survival mechanisms in embryos. In addition, correct regulation of the RNA binding protein, SUP-26, a *mir-35* family target, is needed for survival in chronic hypoxia. The identification of the full mRNA target repertoire of these miRNAs will reveal the miRNA-regulated network of hypoxic survival mechanisms in *C. elegans*.

Oxygen (O_2_) is crucial for cellular survival. Its availability has had an enormous impact on shaping life in the variety of ecosystems, and many organisms have evolved to live and reproduce under the ambient O_2_ concentration (21%). The correct level of O_2_ in tissues during embryogenesis is particularly important for the successful execution of a variety of developmental events^1^. However, abnormally low O_2_ levels (hypoxia) during development can lead to permanent defects and even premature termination of embryogenesis^2^,^3^. Therefore, embryos have developed molecular strategies to cope with inherent as well as exogenous hypoxic conditions^4^,^5^. The core hypoxic pathway relies on stabilization of the hypoxia-inducible factor HIF-1, which regulates the transcription of many protein-coding genes and microRNAs (miRNAs) responsible for the hypoxic response^6^,^7^. In addition, *hif-1*-independent hypoxic pathways have been identified in different models^8^,^9^. This suggests that other *hif-1* independent pathways may exist to regulate aspects of the hypoxic response.

miRNAs are small (18–25nt) non-coding RNA molecules that were first discovered in *Caenorhabditis elegans*^10^. They function mainly as negative post-transcriptional regulators by binding to the 3’ untranslated region (UTR) of target mRNAs^11^. miRNAs are grouped in distinct families that share the same seed sequence, which is an important determinant for mRNA target specificity. Therefore, members of a miRNA family can conceptually act in a redundant manner. To date, miRNAs have been implicated in a range of developmental paradigms in *C. elegans*^10^,^12^,^13^,^14^, in addition to stress-related phenomena such as the regulation of lifespan^5^,^15^,^16^,^17^. Moreover, miRNAs^18^ and other non-coding RNA species^19^ function in the hypoxic response^20^. Their active turnover and ability to simultaneously regulate more than one target are properties that make miRNAs potentially useful stress response molecules^21^,^22^. Although they appear to regulate the majority of mRNAs in a cell^23^, information is often lacking on their specific role in different stress responses. We therefore sought to identify miRNAs that are important for the regulation of the hypoxic response in *C. elegans*.

We screened 40 *C. elegans* miRNA mutants covering 22 miRNA families for the ability to control embryonic survival in hypoxia. We found that loss of 14 miRNA families caused embryos to be either sensitive or resistant to hypoxic exposure. Focusing on the *mir-35* family, we found that expression of all members of this family is induced in embryos exposed to hypoxia in a *hif-1*-independent manner. The *mir-35* family is required for hypoxic survival as deletion mutants that remove *mir-35–41* exhibit >90% embryonic lethality under hypoxia. Finally, we identified *sup-26*, which encodes an RNA binding protein, as a target of the *mir-35* family that is, at least in part, required for embryonic survival in chronic hypoxia. Our study is the first to offer insights into miRNA regulation of the embryonic hypoxic response in *C. elegans* and further work will identify targets of these miRNAs that together provide appropriate survival mechanisms in hypoxic conditions.

## Results and Discussion

### Multiple miRNAs are important for the embryonic hypoxic response

In an effort to identify miRNAs important for the control of hypoxic responses in *C. elegans* embryos, we screened a collection of mutants that lack individual or multiple miRNAs for embryonic survival under hypoxia (Figure S1, Table S1 and Table S2). We exposed semi-synchronized populations of embryos to 0.5% O_2_ for 24 hours at 20 ^o^C and after recovery for 24 hrs at 21% O_2_ embryos were scored for survival (Figure S1). To exclude phenotypes caused by background mutations, we outcrossed the mutants and used multiple alleles or strains where possible. Using this approach, we were able to identify multiple gene families that regulate the ability of embryos to survive a hypoxia insult (Table S1). Loss of certain miRNAs or miRNA families led to hypoxia sensitivity (*mir-2*, *mir-35, mir-44*, *mir-49*, *mir-51*, *mir-60*, *mir-63* and *mir-67*) and others to hypoxia resistance (*let-7*, *mir-58*, *mir-67*, *mir-79, mir-237*, *mir-246*, *mir-359*). Finally, we found that 20 miRNA mutant strains exhibited similar embryonic survival rates to wild type animals (Table S2). These data suggest that multiple miRNAs are required for *C. elegans* embryos to respond appropriately to hypoxia.

### The *mir-35–41* family regulates the embryonic response to hypoxia

**Figure 1:**
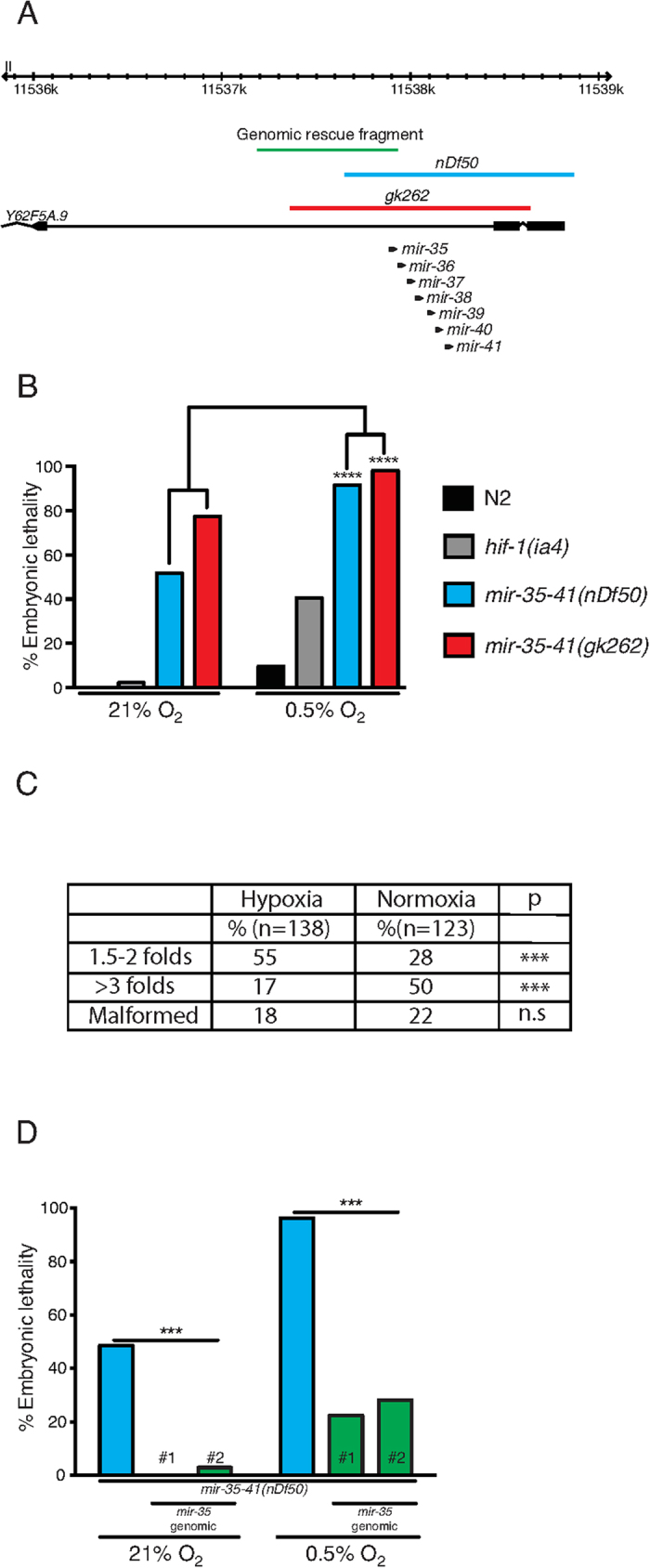
The mir-35 family is required for embryonic hypoxic survival. (**A**) *mir-35–41* locus, mutant alleles and genomic rescue fragment. *nDf50* (blue) and *gk262* (red) alleles remove the entire *mir-35–41* locus and part of the *Y62F5A.9* gene. The genomic rescue fragment used in (**D**) is marked in green, which includes a 602 bp upstream region and the *mir-35* hairpin. (**B**) At 21% O_2_, two *mir-35–41* mutant alleles, *nDf50* and *gk262,* exhibit 50% and 75% embryonic lethality respectively, whereas, wild type and *hif-1(ia4)* mutant embryos exhibit minimal lethality. At 0.5% O_2_, both *mir-35–41* mutants approach 100% embryonic lethality (n = 176–242), whereas wild type and *hif-1(ia4)* mutant embryos exhibit 10% (n = 974) and 40% (n = 1132) lethality respectively. These data partially overlap with Table S1. (**C**) An increased percentage of *nDf50* mutant embryos die younger in hypoxia than in normoxia. ‘Malformed’ refers to embryos with severe defects in their overall structure that do not permit stage identification. (**D**) Normoxic and hypoxic lethality of *nDf50* mutant embryos is rescued by transgenic expression of *mir-35*. The sequence used to rescue *mir-35* is shown as a green line in (**A**). n = 96–125. # refers to independent transgenic lines. Contingency table values are presented and Fischer exact test applied for statistical evaluation. ***≤0.001, ****≤0.0001. n.s. = not significant.

We focused our studies on the *mir-35–41(nDf50)* mutant strain due to its high sensitivity to hypoxia (Fig. 1 and Table S1). The mutant strain carrying the *nDf50* deficiency lacks 7 (*mir-35–41*) of the 8 members of *mir-35* family (Fig. 1A). The embryonic lethality observed in this mutant under ambient O_2_ concentration (21% O_2_) at 20 ^°^C is approximately 50%, in agreement with previous reports^24^, however, lethality is increased to over 90% when freshly-laid embryos are exposed to 0.5% O_2_ for 24 hrs at 20 ^°^C (Fig. 1B). Hypoxia sensitivity was also phenocopied in the independently isolated *mir-35–41(gk262)* mutant strain (Fig. 1A–B). A higher percentage of *nDf50* mutant embryos die younger in hypoxia than in normoxia, implying that hypoxia affects development at an earlier stage in this mutant (Fig. 1C). Indeed, when we subjected older embryos (6–9 h old) to hypoxia we observed significantly lower embryonic lethality compared to when we subjected early embryos (0–3 h old) (Figure S2A). The similarity between the elevated *nDf50* embryonic lethality we found in hypoxia and the known high lethality of this mutant in high temperature^24^ may reflect common components of hypoxia and heat stress such as challenges in protein folding and stability and induction of heat shock proteins^25^. However, when we subjected both *mir-35–41* deletion mutant strains to 2 g/l sodium sulfite, which mimics hypoxic stress^26^, we also observed a significant increase in embryonic lethality (Figure S3A) indicating that *mir-35* family has a specific role in survival of embryos in hypoxia.

The *mir-35–41* cluster is located within an intron of a worm specific gene (*Y62F5A.9*), and both *nDf50* and *gk262* lesions affect exonic sequences of this gene (Fig. 1A). We therefore performed rescue experiments by expressing either *mir-35* alone or the entire *mir-35–41* cluster under the control of *mir-35*-locus upstream sequence located within the intron of its host gene (Fig. 1 and Figure S2B–C). We rescued embryonic lethality in both normoxic and hypoxic conditions using these strategies (Fig. 1D and S2C). This indicates that the *mir-35* family is required for the hypoxic response of embryos and implies that a single *mir-35* family member can rescue phenotypes of *mir-35–41* mutant animals, in accordance to previous reports^24^,^27^,^28^. However, we wished to exclude the possibility that the hypoxia-induced embryonic lethality of *mir-35–41* mutant embryos is due to general sensitivity of strains that exhibit a high embryonic lethality in ambient O_2_ conditions. Therefore, we analyzed the hypoxic response of mutants of three unrelated genes (*plk-1*, *spd-2* and *mex-1*) that exhibit high normoxic embryonic lethality. We exposed embryos of these mutant strains to 0.5% O_2_ and did not observe any significant hypoxia-induced enhancement of embryonic lethality (Figure S3B, Table S3). These data point to a specific role for the *mir-35* family in the embryonic hypoxic response.

### Hypoxic induction of the *mir-35* family is independent of HIF-1

**Figure 2:**
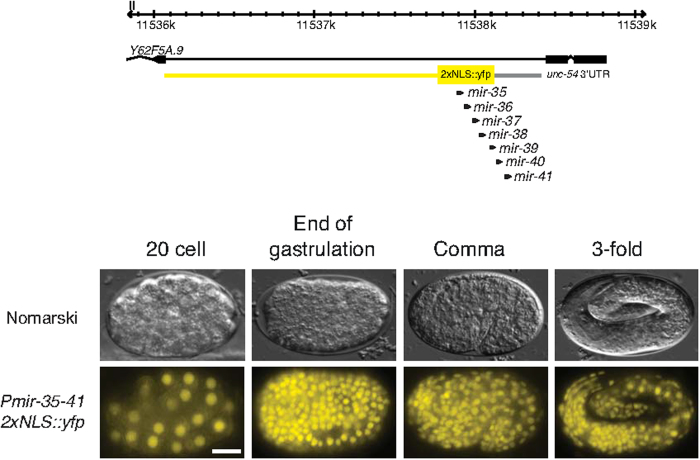
The mir-35–41 promoter drives ubiquitous expression in the embryo. A *mir-35–41*^*prom*^*::2xNLS::yfp* transcriptional reporter drives expression throughout the embryo from the 20 cell stage. The region used to drive *yfp* expression is shown in yellow on the genomic view (top). Upper panels are Nomarski micrographs and bottom panels are fluorescence images of the same embryos. Anterior is to the left. Scale bar 10 μm.

The *mir-35* family is predominantly expressed during embryogenesis^24^. We confirmed this expression pattern by constructing a transgene using a 602 bp intronic promoter driving expression of *yfp* fused to a nuclear localization signal (NLS) (Fig. 2). This promoter region was sufficient to rescue the *nDf50* hypoxic phenotype when driving the *mir-35–41* cluster (Figure S2). We detected YFP expression in virtually all cells starting from ∼20 cell embryos and persisting throughout mid- and late embryogenesis (Fig. 2). We also detected expression in L1 animals but the signal drops in later larval stages. Thus, the rescuing *mir-35* intronic promoter drives expression throughout embryogenesis in most, if not all cells.

**Figure 3:**
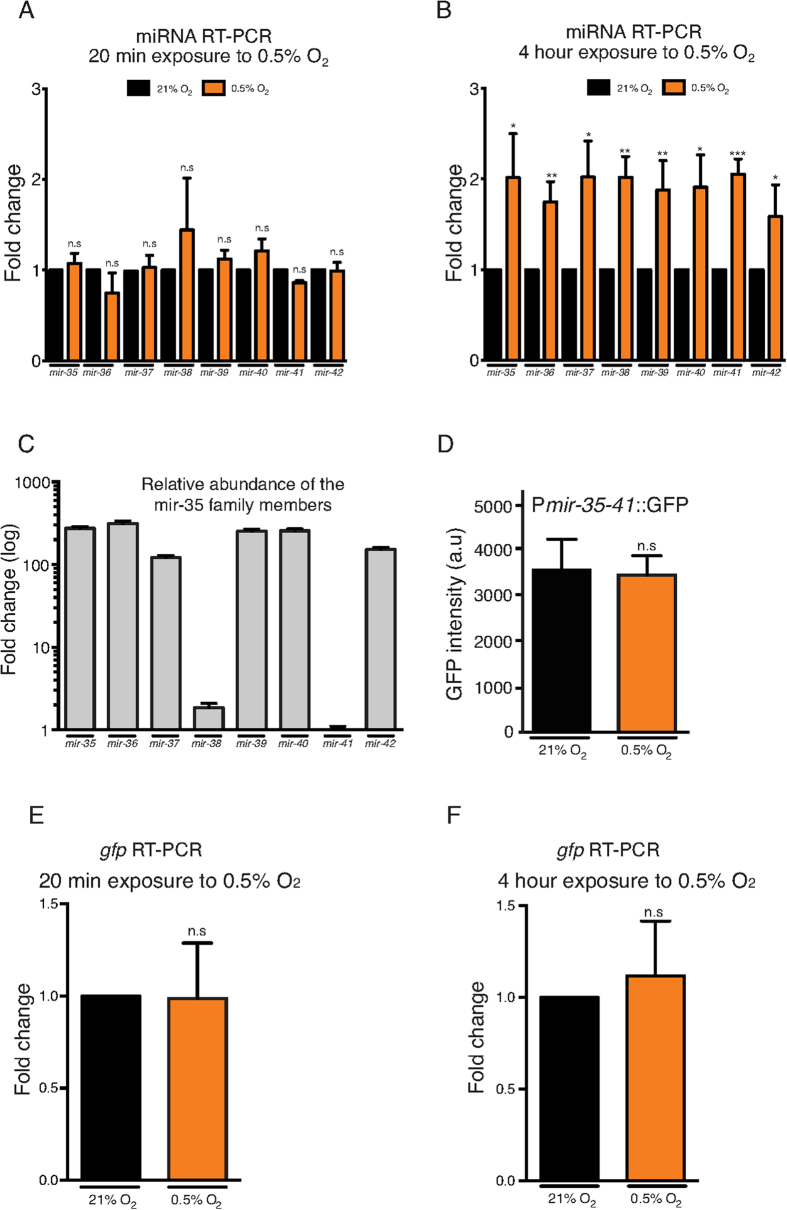
Expression of the mir-35 family is regulated by chronic hypoxia. (**A–B**) qRT-PCR showing *mir-35* family member expression levels in wild type embryos exposed to 21% O_2_ (black bars) or 0.5% O_2_ (orange bars) for 20 mins (**A**) or 4 hrs (**B**). The level of normoxic expression was set to 1 for each of the three repetitions. (**C**) Graphical representation of *mir-35* family member abundance in normoxia. *mir-38* and *mir-41* are less abundant than the other family members, even from those miRNAs transcribed in the same cluster. Values on the graph are logarithmic functions with base 10 of the fold change value for each miRNA. *mir-41* showed the lowest relative abundance and was arbitrarily set as the value 1. Primer efficiencies are 116%, 118%, 116%, 113%, 93%, 93%, 109% and 118% for each respective miRNA. (**D**) Intensity of GFP expression driven by the *mir-35–41* promoter is unaffected by 4 hrs of hypoxic exposure. The transgene used is *wwIs8[pmir-35–41::GFP + unc-119(+)]*. (**E**–**F**) *gfp* transcription, driven by the *mir-35–41* promoter in wild type embryos exposed to 21% O_2_ (black bars) or 0.5% O_2_ (orange bars) for 20 mins (**E**) or 4 hrs (**F**). Data are presented as means of at least 3 independent repetitions and error bars represent ± SD. Students t-test was used to assess for statistical significance. *p ≤ 0.05, **≤0.01, ***p ≤ 0.001, ****≤ 0.0001, n.s. = not significant.

We have shown that the *mir-35* family is required for hypoxic survival of embryos and is expressed throughout embryogenesis. Next, we asked whether the level of each *mir-35* family member is regulated by hypoxia. We therefore subjected early embryos to 0.5% O_2_ for 20 mins or 4 hrs, to test for acute and chronic responsiveness, and measured the levels of the mature sequence of each family member by quantitative real-time PCR (qRT-PCR). We found that the expression of the mature sequence of all eight members of the *mir-35* family was induced by approximately 2-fold after 4 hrs but not after 20 mins (Fig. 3A–B). Whereas, expression of the *Y62F5A.9* host gene is not induced (Figure S4C) and expression of all three reference miRNAs (*mir-34, mir-86* and *mir-1829c*) is stable in these conditions (geNorm M value <0.5, Coefficiency of variation <0.2). Interestingly, the expression of predicted primary and precursor forms of the *mir-35–41* locus measured by a previously described qPCR-based method^29^, using primers flanking the middle member of the cluster (*mir-38),* is decreased after 4h in hypoxia (Figure S4D). This suggests post-transcriptional regulation of this locus upon hypoxia. As previous studies in *C. elegans* have shown that HIF-1 stabilization and general downregulation of translation are important requirements for animals to survive hypoxic insults^25^,^30^,^31^, such regulation of the *mir-35* family could be dependent on HIF-1 transcription. Therefore we subjected *hif-1(ia4)* mutant embryos to 4 hrs of 0.5% O_2_ and quantified induction of the *mir-35* family. We still detected induction of *mir-35–42* indicating that this is independent of HIF-1 (Figure S4A). Previous work has shown that *C. elegans* embryos are more sensitive to hypoxia when they are exposed directly to low O_2_ rather than *in utero*^32^. This prompted us to ask whether hypoxia-induced *mir-35–42* induction may differ *in utero*. We therefore, subjected late L4 animals to 0.5% O_2_ for 15 hrs and developing embryos were subsequently extracted from the gonad. Using qRT-PCR analysis we observed an approximate 4-fold induction in expression of all *mir-35* family members except for *mir-38* and *mir-41* (Figure S4B). These data suggests that post-transcriptional mechanisms within the mother regulate the differential expression of the *mir-35* family under hypoxic stress. Taken together, these results show that embryonic expression of the *mir-35* family is induced by hypoxia in a *hif-1*-independent manner and that *mir-38* and *mir-41* are differentially regulated by hypoxia *in utero*.

During our qRT-PCR analysis, we observed additional evidence that the *mir-35* cluster is potentially post-transcriptionally regulated. We observed that the expression levels of the mature miRNAs differ between members of the *mir-35* family. In particular, based on the measured Cq values, mature *mir-38* and *mir-41* are much less abundant than the other members, 117- and 215-fold less abundant respectively (in accordance with previous reports^33^) when compared to the average abundance of the other 6 members (Fig. 3C). This observation further supports the possibility that *mir-38* and *mir-41* are differentially processed after transcription of the locus.

We next asked whether hypoxic induction of the *mir-35* family is due to enhanced transcription of the *mir-35–41* locus. To answer this question, we used a strain carrying an integrated *gfp* transgene driven by genomic region upstream of the *mir-35* locus^34^. We subjected embryos of this strain to 0.5% O_2_ for 4 hrs and found that the intensity of *mir-35–41-*promoter driven GFP was similar to that of embryos cultivated in normoxia (Fig. 3D). To confirm these data at the RNA level we subjected the same strain to 0.5% O_2_ for 20 mins or 4 hrs and found that the level of *gfp* mRNA is not elevated in hypoxia in either condition (Fig. 3E–F). Taken together, these results suggest that the induction of the *mir-35* family in hypoxia is controlled by a post-transcriptional mechanism such as increased protection by RNA binding proteins^35^,^36^.

### *sup-26* is a potential *mir-35–41* direct target

Although the involvement of *mir-35* family in embryonic development has been observed in the past^24^,^37^, there is a lack of information as to the downstream regulatory targets of these miRNAs during embryogenesis. Using miRNA target prediction logarithms (mirSOM, TargetScan) we extracted a list of potential direct targets of the *mir-35* family. We screened this list for genes that are potentially related to hypoxia based on published literature. We noticed that the human homolog (RBMS1) of one of the candidates, namely SUP-26, is involved in the brain ischemic response^38^. The 3’UTR of *sup-26* contains a sequence complementary to the seed sequence of all the *mir-35* family members. In addition, SUP-26 co-purifies with *mir-35-42* in embryos as part of miRISC^39^. SUP-26 is an RNA binding protein that acts in the sex determination pathway in *C. elegans*^40^.

**Figure 4:**
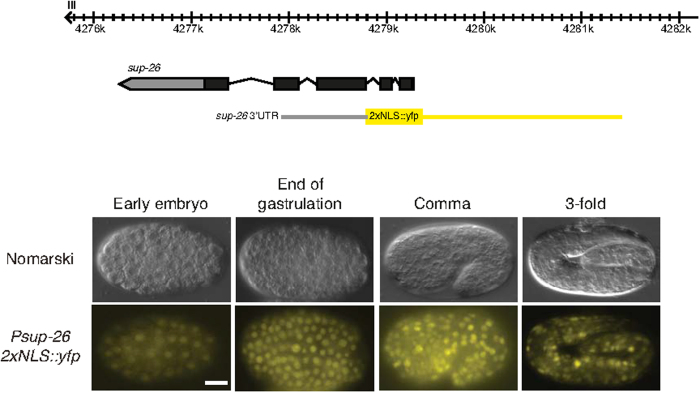
The sup-26 promoter drives ubiquitous expression in the embryo. A *sup-26*^*prom*^*::2xNLS::yfp* transcriptional reporter drives expression throughout the embryo from the 80 cell stage. Region used to drive YFP expression is shown in yellow on the genomic view (top). Upper panels are Nomarski micrographs and bottom panels are fluorescence images of the same embryos. Anterior to the left. Scale bar 10 μm.

As a potential target of the *mir-35* family one would expect *sup-26* to be expressed during embryogenesis, the period at which the *mir-35* family predominates. We therefore generated a transgene using the *sup-26* promoter to drive nuclear-localized YFP followed by the native *sup-26* untranslated region (UTR). We detected ubiquitous expression of YFP throughout embryogenesis indicating that *sup-26* is expressed in a common temporal and spatial window to that of the *mir-35* family (Fig. 4).

**Figure 5:**
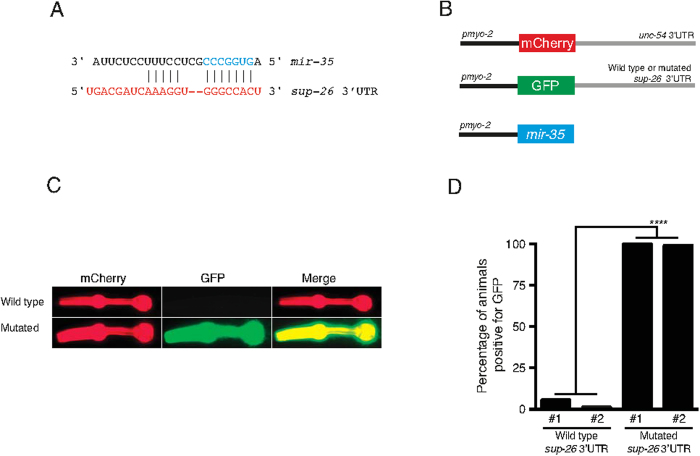
sup-26 is a mir-35–41 target and is required for hypoxic survival. (**A**) The *C. elegans sup-26* 3’ UTR contains a single *mir-35* family binding site (only *mir-35* is shown). The *mir-35* seed sequence is shown in blue and the predicted *sup-26* 3’UTR is shown in red. (**B**) Sensor experiment constructs. *mir-35* was expressed in the pharynx together with a RFP reporter controlled by the unregulated *unc-54* 3’UTR and a GFP reporter controlled by the *sup-26* 3’UTR (wild type or *mir-35* binding site mutated). The *mir-35* binding site in the *sup-26* 3’UTR was mutated from CCCGGUG to CCatGgG to prevent binding of *mir-35* family miRNAs. (**C**) Representative picture of the sensor experiment results. *mir-35* downregulates GFP expression (*sup-26* 3’UTR) and not the RFP sensor (control 3’UTR). Regulation via the *sup-26* 3’UTR is dependent on the *mir-35* binding site. (**D**) Quantification of the sensor experiment results. n > 50. Fischer exact test was used for statistical evaluation. # refers to independent transgenic lines. ****≤0.0001.

To test the possibility that *sup-26* is a direct *mir-35* target we conducted sensor experiments in a heterologous tissue, the pharynx, after failing to do so in embryos due to specific transgene toxicity (data not shown). We drove expression of *mir-35* in the pharynx in combination with a RFP reporter under the control of the unrelated *unc-54* 3’UTR and a GFP reporter under the control of the *sup-26* 3’UTR (wild type or *mir-35* binding site mutated) (Fig. 5B–D). We found that *mir-35* robustly downregulates GFP expression (*sup-26* 3’UTR) and not the RFP sensor (control 3’UTR) (Fig. 5B–D). Further, regulation via the *sup-26* 3’UTR is dependent on the predicted *mir-35* binding site (Fig. 5B–D) strongly suggesting a direct interaction between *mir-35* and the *sup-26* 3’UTR. Interestingly, a genetic interaction between these two molecules was recently observed independently by the Ambros group^41^.

Finally, we asked whether *sup-26* plays a role in the hypoxic response of embryos as one may expect as a potential *mir-35* family target. We therefore subjected two independent *sup-26* loss-of-function alleles, *gk403* and *gk426*, to embryonic hypoxia (Figure S5). Both mutants exhibited the wild type level of embryonic lethality when exposed to 0.5% O_2_ for 24 hrs, however, when we exposed embryos to chronic hypoxia (45 hrs), we observed a significant increase in embryonic lethality (Figure S5 and data not shown). This increase was reduced to the wild type levels when the *sup-26* product was reintroduced to the *sup-26(gk403)* mutant (Figure S5B). These data suggest that *sup-26* plays a role in the embryonic response to hypoxia. However, since reduced SUP-26 levels resulted in embryonic lethality under low oxygen conditions, we hypothesize that the *mir-35* family negatively regulates *sup-26* in order to keep its expression to a physiological level, but not to eliminate its expression, for optimal survival of the embryos in hypoxia. Thus, defective regulation of *sup-26* levels may be one of the causes of hypoxic lethality exhibited by loss of the *mir-35* family. Other *mir-35* target genes are also very likely to be involved in this process since the embryonic lethality of the *sup-26* mutants is not significantly increased by the milder oxygen conditions (0.5% for 24 hrs).

miRNAs, such as *miR-210* were previously shown to play a pivotal role in hypoxic response in mammals and other organisms by regulating key factors and also being regulated by *hif-1*^42^. Our study offers insights into the role of additional miRNA families in hypoxia in a highly attractive experimental system, the *C. elegans* embryo, and we hope that further work based on these findings will extend our current knowledge on the role of miRNAs in protecting tissues when challenged with decreased oxygen tension.

## Methods

### Strains used

All strains used in this study are listed in Table S4.

### Molecular biology and transgenic lines

The oligonucleotides sequences used in this study are listed in Table S5.

### Embryonic lethality in hypoxia

See Supplementary information for details. Briefly, plates containing embryos were placed for 24 hrs at 0.5% O_2_ (nitrogen balanced). After 24 hrs recovery in ambient O_2_ conditions, embryonic lethality was scored (Figure S1). For the *in utero* hypoxia experiment, mid-L4 animals were placed in 0.5% O_2_ for 15 hrs and eggs were removed from mothers by bleaching followed by immediate RNA extraction.

### mRNA isolation and qRT-PCR analysis

miRNA qPCRs were performed as described in^42^. All qRT-PCR primers were designed using the on line tool described in^43^. See Supplementary information for further details.

### Statistical analysis

See Supplementary information.

### Microscopy

Animals were anaesthetized on 5% agarose pads using 20 mM NaN_3_. Images were taken using a fluorescence microscope and the Zen software (Zeiss, AXIO Imager M2).

## Additional Information

**How to cite this article**: Kagias, K. and Pocock, R. microRNA regulation of the embryonic hypoxic response in *Caenorhabditis elegans*. *Sci. Rep.* **5**, 11284; doi: 10.1038/srep11284 (2015).

## Supplementary Material

Supplementary Information
