# Supplementary material for: microRNA regulation of the embryonic hypoxic response in Caenorhabditis elegans
Source: Sci Rep. 2015 Jun 11;5:11284. doi: 10.1038/srep11284 (PMC4462753; doi:10.1038/srep11284)
Supplement: Supplementary Information [file srep11284-s1.pdf]

microRNA regulation of the embryonic hypoxic response in *Caenorhabditis elegans*

Konstantinos Kagias[1] and Roger Pocock[1,2*]

[1]Biotech Research and Innovation Centre (BRIC), University of Copenhagen, Ole Maaløes Vej 5, Copenhagen, Denmark

[2]Department of Anatomy and Developmental Biology, Faculty of Biomedical and Psychological Sciences, Monash University, Clayton, Victoria, Australia.

SUPPLEMENTARY EXPERIMENTAL PROCEDURES

Strains

Strains were grown on nematode growth media (NGM) agar at 20°C on the *Escherichia coli* OP50 strain using standard growth conditions [1]. Transgenic animals were created as described in [2]. All the experiments were conducted at 20°C. A list of strains used in this study is provided in Table S3.

Molecular biology

For the *mir-35(ndf50)* mutant rescue, we used a 642bp upstream promoter region to drive either the full *mir-35-41* cluster (primers KK163/164) or *mir-35* alone (primers KK163/171). The PCR products were injected at 3ng/μl together with 5ng/μl of *elt-2::gfp* as co-injection marker. For the *Pmir-35-41 2xNLS::yfp* construct, we used a 602bp upstream promoter region of *mir-35-41* cluster (primers KK240/241) inserted upstream of 2xNLS::YFP using BamHI/HindIII. This construct was injected at 3ng/μl together with 5ng/μl of *elt-2::gfp* as co-injection marker. For the *Psup-26::2xNLS::yfp*, a 2080bp fragment upstream of the *sup-26* start codon was used (primers KK238/239) and inserted upstream of *2xNLS::YFP* using BamHI/HindIII. In this construct, the *unc-54* 3'UTR was substituted by the *sup-26* 3' UTR (primers KK243/244) using ApaI/EcoRI. This construct was injected at 50ng/μl. For the *sup-26* sensor experiment, the *myo-2* promoter was used to drive the *mir-35* hairpin and the fluorescent reporters upstream of the respective 3'UTRs. For the *sup-26* rescue, a previously described transgene [3] was used.

Embryonic lethality in hypoxia

Approximately 20 L4 stage animals were placed in a plate seeded with a 300 μl drop of OP50 LB culture, and 24 hrs later were moved as young adults to a new plate, seeded

with a 300 µl drop of OP50, to lay eggs for 3h (egg pulse). Next, mothers were removed and the plates containing the eggs were placed for 24 hrs in an air-proof chamber (Billups-Rothenberg) filled with 0.5% O_2 and 99.5% nitrogen. After this time, the plates were left to recover for another 24 hrs in ambient O_2 conditions and embryonic lethality was scored and calculated by counting unhatched eggs and larvae (Figure S1). For the *in utero* hypoxia experiment, staged L4 animals were placed in 0.5% O_2 for 15 hrs and eggs were removed from mothers by bleaching followed by immediate RNA extraction. All the strains in the screen were scored at least 2 independent times, most of them more than 3 times. N2 and *hif-1* mutant animals were included as controls in each assay to assess for effective hypoxic conditions. Scorings were pooled for each strain.

mRNA isolation and qRT-PCR analysis

Embryos obtained by bleaching: Staged early young adult animals were bleached and the eggs obtained were subjected to the different hypoxic conditions. RNA was isolated using standard Trizol based methods [4].

Total cDNA was obtained using TaqMan Reverse Transcription Reagents (Invitrogen, Cat. No: N8080234). qRT-PCR reactions were performed in triplicates on a LightCycler 480 System (Roche) using the 33 Maxima SYBR/ROX qPCR Master Mix (Fermentas, Cat. No: K0221) and the results were analyzed using qBase from Biogazelle. Error bars represent the SEM of at least 3 independent sets of samples. Three qRT-PCR reference genes were used - *mir-34*, *mir-86* and *mir-1829c* for miRNAs and *cdc-42*, *pmp-3* and *Y45F10D.4* for mRNAs. The stable expression of these genes was tested in each experiment using the geNORM logarithm [5]. Results presented as 'fold change' values normalized to the arbitrary value 1 given to a defined sample (e.g. normoxic sample).

For the detection of primary and precursor species, the procedure described in [6] was followed. This method is used to measure both primary and precursor miRNAs without however being able to distinguish the two. The RT reactions were performed using gene specific primers. The RT primer for the *mir-35-41* cluster was designed to bind at the 5' end of the suspected polysystronic RNA. qPCR was performed using primers designed to bind at the stem region of the *mir-38* hairpin.

Statistical analysis

Data of embryonic lethality phenotype are presented as contingency table values derived from 2, 3 or more independent assay repetitions. Fischer exact test was used for statistical analysis of these data. qPCR data are presented as means of at least 3 independent repetitions and error bars represent ± SD. Students t-test was used in this case to assess for statistical significance using Prism 5.0d (GraphPad Software, Inc.). P-values was added on the graphs as *$p \leq 0.05$, **$p \leq 0.01$, ***$p \leq 0.001$, ****$p \leq 0.0001$, n.s. = not significant.

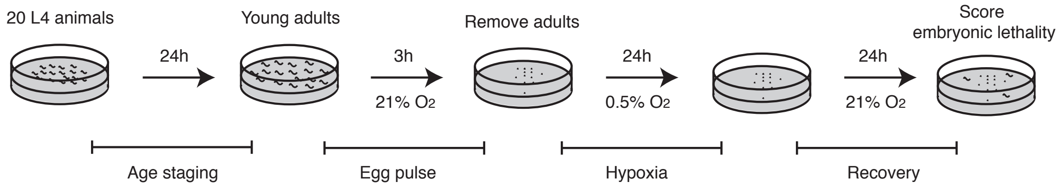

Figure S1. Embryonic hypoxic survival assay

Schematic of the experimental pipeline used for screening and scoring hypoxic survival of miRNA mutants. 20 L4 animals were picked from a well-fed, uncrowded, mixed worm population and placed on a freshly seeded OP50 plate. 24 hours later these now 1-day adult animals were transferred to a new freshly seeded OP50 plate and allowed to lay eggs for 3 hours (egg-pulse). After removal of the mothers these eggs were placed in 0.5% O_2 for 24 hours (hypoxia) and following a 24-hour recovery time the embryos were scored for survival. All experiments were performed at 20°C.

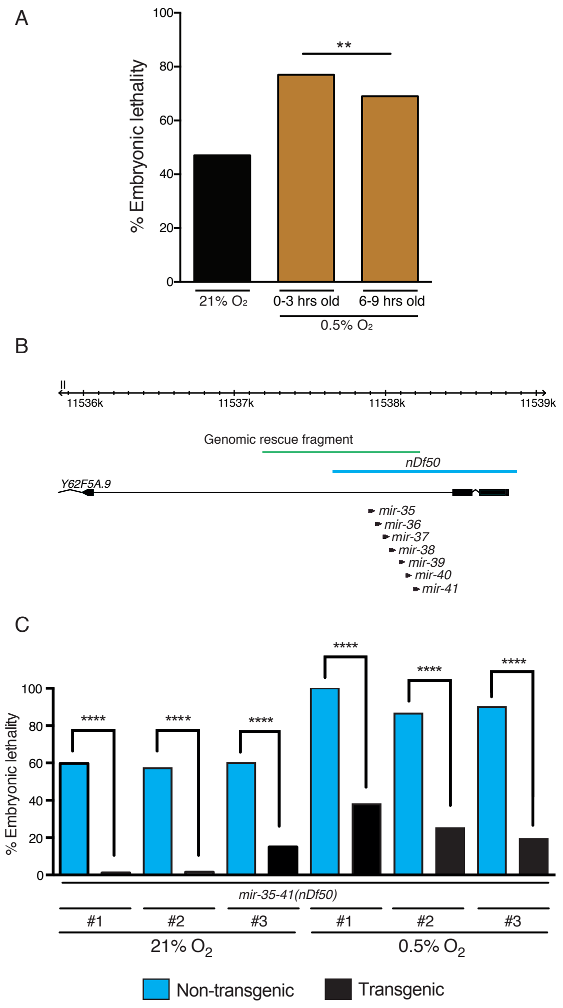

Figure S2. Further characterization and transgenic rescue of *mir-35-41(nDf50)* embryonic lethality

(A) Old embryos (6-9h post egg-laying) subjected to hypoxia show significantly lower embryonic lethality compared to early embryos (0-3h post egg-laying). n=250-525. Fischer exact test was used for statistical analysis. **≤ 0.01.

(B) The *nDf50* (blue) deficiency removes the entire *mir-35-41* locus and part of the worm specific gene *Y62F5A.9*. The genomic rescue fragment used in (C) is marked in green, which includes a 602 bp upstream region and the *mir-35-41* cluster.

(C) Normoxic and hypoxic lethality of *mir-35-41(nDf50)* mutant embryos is rescued by transgenic expression of the *mir-35-41* cluster. The sequence used to rescue *mir-35* is shown in (A). n=20-423. ****≤ 0.0001. # refers to independent transgenic lines.

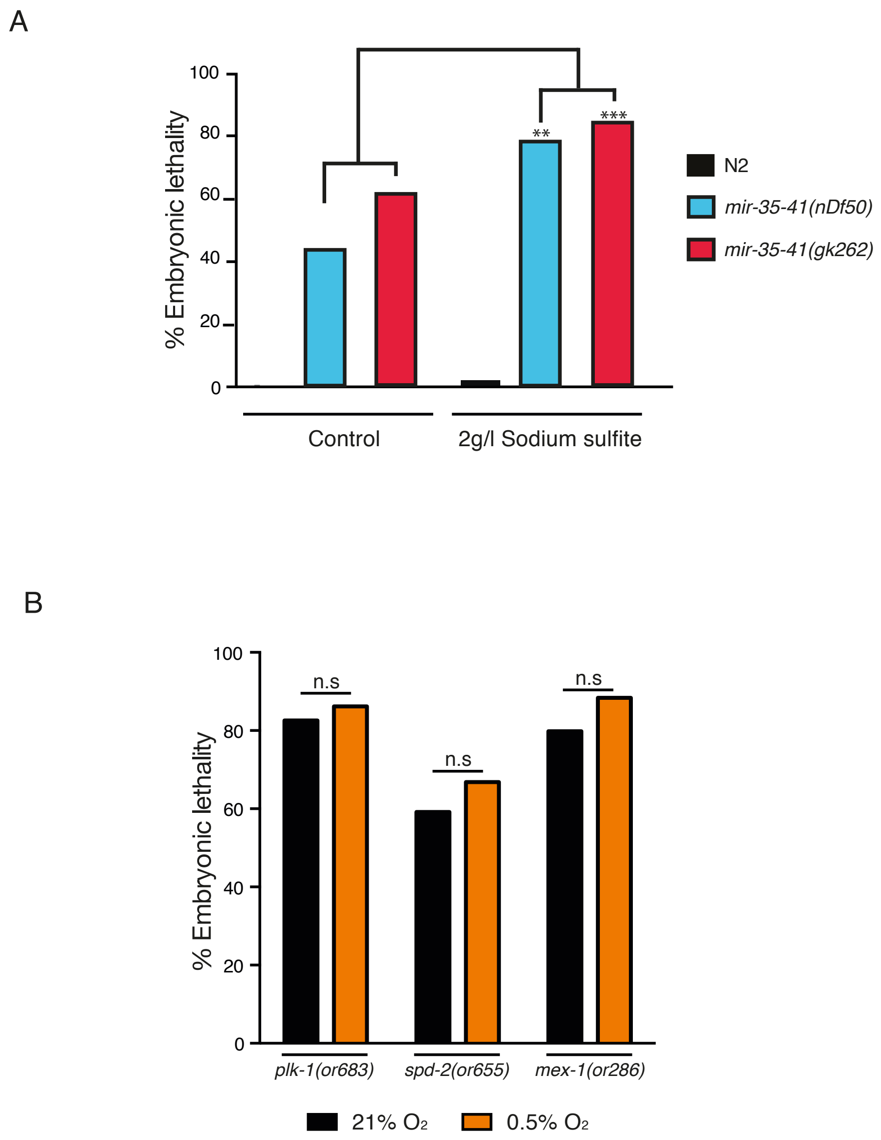

Figure S3. *mir-35-41* deletion mutant strains are sensitive to sodium sulfite and independent mutant strains with high embryonic lethality do not exhibit hypoxia sensitivity

(A) Both *mir-35-41* deletion mutant strains show a significant increase in embryonic lethality when subjected to 2g/l sodium sulfite, a compound that mimics hypoxic stress. (B) The normoxic embryonic lethality of three different mutants - *plk-1(or683)*, *spd-2(or655)* and *mex-1(or286)* does not significantly increase under hypoxia. Embryos of each strain were treated as described in Figure S1. 21% O_2 (black bars) or 0.5% O_2 (orange bars). n=75-450. n.s. - not significant.

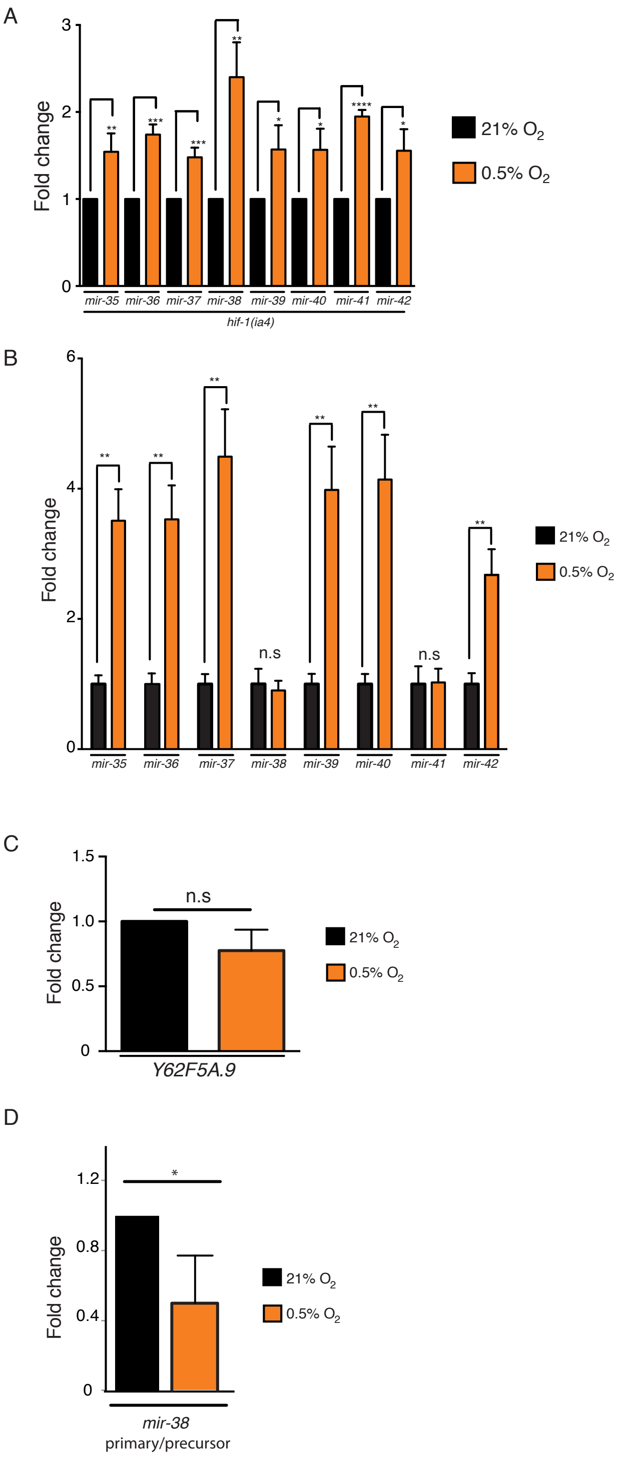

Figure S4. qRT-PCR analysis

(A) qRT-PCR showing *mir-35* family member expression levels in *hif-1* mutant embryos exposed to 21% O_2 (black bars) or 0.5% O_2 for 4 hrs (orange bars).

(B) qRT-PCR showing *mir-35* family member expression levels in wild type embryos exposed to 21% O_2 (black bars) or 0.5% O_2 (orange bars) for 15 hrs *in utero*.

(C) The *mir-35-41* host gene, Y62F5A.9, is not induced by hypoxia. n.s. - not significant.

(D) The expression of primary and precursor forms of the *mir-35-41* locus measured by qPCR using primers flanking either the *mir-35* or *mir-38* hairpin does not change in hypoxia.

Students t-test was used for statistical significance. *p≤ 0.05, **≤ 0.01, ***p≤ 0.001, ****≤ 0.0001, n.s. = not significant.

A

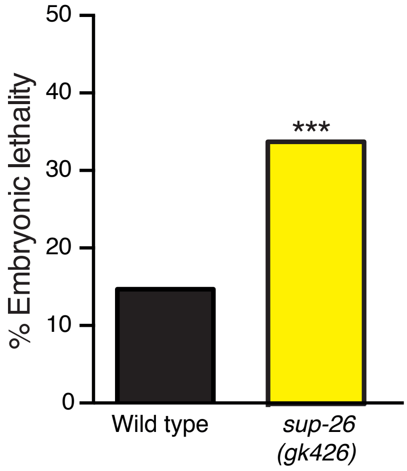

B

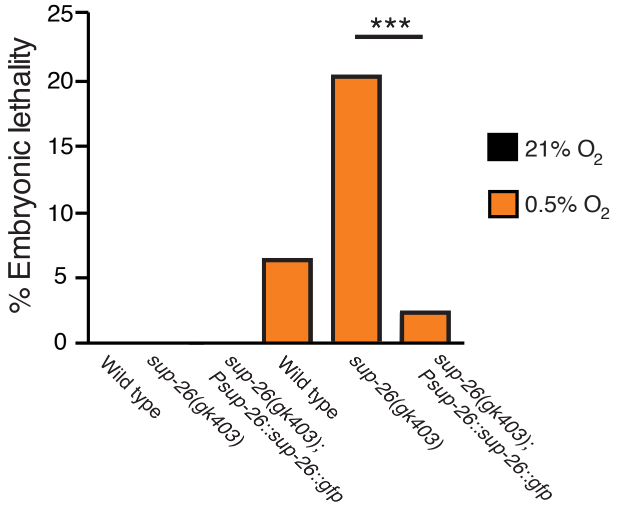

Figure S5. *sup-26(gk426)* embryos are sensitive to hypoxia

(A) Chronic hypoxic exposure (0.5% O_2 for 45h), causes lethality of *sup-26(gk426)* embryos. n=73-470. ***≤ 0.001.

(B) The embryonic lethality of *sup-26(gk403)* mutant animals under chronic hypoxic exposure (0.5% O_2 for 45h) is rescued by the resupply of *sup-26* gene driven by its native promoter. n>50. ****≤ 0.0001.

Table S1. miRNA mutants exhibiting hypoxic sensitivity or resistance phenotypes

Controls and alleles	Strain	Number of Outcrosses	Genotype	Percentage Embryonic Lethality (n) Hypoxia	Percentage Embryonic Lethality (n) Normoxia	p value (compared to wt)	p value (hypoxia vs normoxia)
**Controls**	N2		Wild type	10 (974)	0 (n>500)		
	OH8125	11	*hif-1(ia4)*	41 (1132)	2 (n=276)	****	****
**Sensitive to hypoxia**							
***mir-35* family**	MT14119	9	*mir-35-41(nDf50)*	91 (242)	51 (n=327)	****	****
	VC514		*mir-35-41(gk262)*	98 (176)	77 (n=133)	****	****
***mir-44* and *mir-2* families**	MT17431		*mir-42-44(nDf49); mir-61&mir-250(nDf59); mir-247(n4505)*	25 (253)	0 (n=26)	****	****
	MT14875		*mir-61&mir-250(nDf59)*	34 (138)	0 (n>100)	****	****
	MT17676		*mir-45(n4280); mir-61&mir-250(nDf59); mir-247(n4505)*	36 (90)	0 (n>100)	****	****
	MT13433		*mir-45(n4280)*	29 (76)	0 (n>100)	****	****
***mir-49* and *mir-67* families**	KB12	6	*mir-67(n4899); mir-83(n4638)*	14 (417)	0 (n>100)	**	****
***mir-51* family**	MT17136		*mir-51&mir-53(nDf67); mir-54-56(nDf58)*	38 (42)	2 (n=54)	****	****
***mir-60* family**	MT16471		*mir-60(n4947)*	15 (273)	0 (n>100)	**	****
	RJP1030	3	*mir-60(n4947)*	22 (46)	0 (n>100)	*	****
***mir-63* family**	MT16494	2	*mir-229&mir-64-66(nDf63)*	32 (169)	0 (n>100)	****	****
	RJP999	5	*mir-229&mir-64-66(nDf63)*	19 (296)	0 (n>100)	****	****
**Resistant to hypoxia**							
***let-7* family**	RJP1015		*lin-58(n4097); mir-84(n4037)*	0 (39)	0 (n>100)	*	n.s
***mir-58* family**	MT13949		*mir-80(nDf53)*	3 (515)	0 (n>100)	****	n.s
	RJP936	3	*mir-80(nDf53)*	2 (241)	0 (n>100)	****	n.s
***mir-67* family**	KB10	6	*mir-67(n4899)*	3 (95)	0 (n>100)	*	n.s
***mir-79* family**	MT14448	2	*mir-79(n4126); mir-75(n4472)*	4 (357)	0 (n>100)	***	*
***mir-237* family**	MT13653		*mir-237(n4296)*	2 (117)	0 (>100)	**	n.s
***mir-246* family**	MT15020		*mir-246(n4636)*	4 (565)	0 (n>100)	***	n.s
***mir-359* family**	MT14673		*mir-359(n4540)*	4 (384)	0 (n=30)	***	n.s

Table S1. miRNA mutants exhibiting hypoxic sensitivity or resistance phenotypes

Description of the embryonic hypoxia assay is depicted in Figure S1. *p≤ 0.05, **≤ 0.01, ***p≤ 0.001, ****≤ 0.0001 refer either to the difference in hypoxic embryonic lethality between each strain and wild type (‘compared to wt’ column) or to the difference in embryonic lethality between normoxia and hypoxia for each strain (‘hypoxia vs. normoxia’ column).

Controls and alleles	Strain	Number of Outcrosses	Genotype	Percentage Embryonic Lethality (n)	p value
**Controls**	N2		Wild type	9.54 (974)	
	OH8125	11	*hif-1(ia4)*	40.54 (1132)	****
**miRNA knockouts with no embryonic hypoxia phenotype**					
***mir-239a* family**	MT15312		*mir-239a & mir-239b(nDf62)*	11 (187)	n.s
	MT16061	4	*mir-238(n4112); mir-239a & mir-239b(nDf62)*	7 (442)	n.s
***mir-44* and *mir-2* families**	MT16309	2	*mir-247 & mir-797(n4505)*	11 (244)	n.s
***mir-78* family**	MT15021		*mir-78(n4637)*	11 (260)	n.s
***mir-51* family**	MT17137		*mir-51(n4473); mir-54-56(nDf58)*	8 (558)	n.s
	MT14767		*mir-54-56(nDf58)*	6 (90)	n.s
	MT12989		*mir-53(n4113)*	10 (673)	n.s
***mir-266* family**	MT13078		*mir-73 & mir-74(nDf47)*	6 (194)	n.s
	RJP929	3	*mir-72(n4130); mir-73 & mir-74(nDf47)*	12 (108)	n.s
	RJP920	3	*mir-72(n4130)*		
***mir-79* family**	MT18037	3	*mir-75(n4472)*	9 (375)	n.s
***mir-233* family**	RJP168		*mir-87(n4104); mir-233(n4761)*	3 (56)	n.s
***mir-256* family**	VC576		*mir-1(gk276)*	3 (76)	n.s
***mir-77* family**	MT16311		*mir-77(n4286)*	12 (535)	n.s
***mir-235* family**	MT17997	2	*mir-235(n4504)*	7 (90)	n.s
***mir-71* family**	MT12993		*mir-71(n4115)*	11 (290)	n.s
***mir-80* family**	MT13954		*mir-81 & mir-82(nDf54)*	5 (344)	n.s
	MT18043	3	*mir-240 & mir-786(n4541)*	5 (373)	n.s
***mir-35* family**	RJP997	3	*mir-42(gk177)*	13 (211)	n.s
***mir-63* family**	MT13016		*mir-229&mir-64(nDf52)*	13 (161)	n.s

Table S2. miRNA mutants exhibiting no hypoxic sensitivity or resistance phenotype

Description of the embryonic hypoxia assay is described in Figure S1. n.s. - not significant, ****≤ 0.0001.

Strain	Genotype	Percentage Embryonic Lethality (n)		p value
		Normoxia	Hypoxia	
EU1441	*plk-1(or683)*	82.6 (75)	86 (87)	n.s
EU1347	*spd-2(or655)*	59.16 (120)	13 (161)	n.s
EU779	*mex-1(or286)*	79.8 (124)	13 (161)	n.s

Table S3. Three mutants with high embryonic lethality do not show significantly enhanced lethality in hypoxia

plk-1(or683), *spd-2(or655)* and *mex-1(or286)* mutants do not exhibit significant increase in embryonic lethality under hypoxia. n.s. - not significant.

Table S4. Strains used in this study

Strain name	Genotype
N2	Wild type
MT14119	*mir-35-41(nDf50)*
VC514	*mir-35(gk262)*
ZG31	*hif-1(ia4)*
RJP1355	*mir-35-41(nDf50); rpEx604(pmir-35::mir-35, elt-2:gfp)*
RJP1357	*mir-35-41(nDf50); rpEx606(pmir-35::mir-35, elt-2:::gfp)*
EU1441	*plk-1(or683)*
EU1347	*spd-2(or655)*
EU779	*mex-1(or286)*
RJP1127	*mir-35-41(nDf50); rpEx544(pmir-35::mir-35-41,elt-2::gfp)*
RJP1128	*mir-35-41(nDf50); rpEx545(pmir-35::mir-35-41,elt-2::gfp)*
RJP1131	*mir-35-41(nDf50); rpEx548(pmir-35-41::mir-35-41,elt-2::gfp)*
RJP1439	*rpEx630(pmir-35::NLS::YFP)*
RJP1537	*rpIs33(psup-26::2NLS::YFP::SUP-26UTR)*
VC1031	*sup-26(gk403)III*
VC901	*sup-26 (gk426)III*
VL413	*wwIs8 [pmir-35-41::GFP + unc-119(+)]*
RJP1731	*rpEx735 (pmyo-2::mCherry unc-54;*<br>*pmyo-2::GFP sup-26 3'UTR; pmyo-2::mir-35 (Line #1)*
RJP1732	*rpEx736 (pmyo-2::mCherry unc-54;*<br>*pmyo-2::GFP sup-26 3'UTR; pmyo-2::mir-35 (Line #2)*
RJP1733	*rpEx737 pmyo-2::mCherry unc-54;*<br>*pmyo-2::GFP sup-26 3'UTR(mut); pmyo-2::mir-35(Line #1)*
RJP1734	*rpEx738 (pmyo-2::mCherry unc-54;*<br>*pmyo-2::GFP sup-26 3'UTR(mut); pmyo-2::mir-35(Line #2)*

Table S5. Primers used in this study

Name	Sequence	Function
KK155f	CGAGTGGACACGTTGCTCTA	nDf50 genotyping
KK155r	ATGGACATTTGGGAGATGGA	nDf50 genotyping
KK159: sup-26qPCRf	CGCTTCGATACTATGTCAAAGG	sup-26 qPCR
KK160: sup-26qPCRr	CATCGAGTGCTTACTCTTCCT	sup-26 qPCR
KK163: mir-35-41fprom	ATCCGTCCACGTCTTCAATC	nDf50 rescue
KK164: mir-35-41r	GGAGGCTCCAGACCTAGG	nDf50 rescue
KK171: mir-35r	AGACACTTTGGATGGTCTAGC	nDf50 rescue
KK174: mir-35fqPCR	CAGTCACCGGGTGGA	*mir-35* qPCR
KK175: mir-35rqPCR	CCAGTTTTTTTTTTTTTTTACTGCT	*mir-35* qPCR
KK176: mir-36fqPCR	CACCGGGTGAAAATTCG	*mir-36* qPCR
KK177: mir-36rqPCR	CCAGTTTTTTTTTTTTTTTCATGC	*mir-36* qPCR
KK178: mir-37fqPCR	GTCACCGGGTGAACAC	*mir-37* qPCR
KK179: mir-37rqPCR	CAGTTTTTTTTTTTTTTTACTGCAAG	*mir-37* qPCR
KK180: mir-38fqPCR	GCAGTCACCGGGAGAA	*mir-38* qPCR
KK181: mir-38rqPCR	TCCAGTTTTTTTTTTTTTTTACTCCA	*mir-38* qPCR
KK182: mir-39fqPCR	TCACCGGGTGTAAATCAG	*mir-39* qPCR
KK183: mir-39rqPCR	GGTCCAGTTTTTTTTTTTTTTTCAAG	*mir-39* qPCR
KK184: mir-40fqPCR	GTCACCGGGTGTACATC	*mir-40* qPCR
KK185: mir-40rqPCR	TCCAGTTTTTTTTTTTTTTTAGCTG	*mir-40* qPCR
KK186: mir-41fqPCR	GTCACCGGGTGAAAAATC	*mir-41* qPCR
KK187: mir-41rqPCR	GGTCCAGTTTTTTTTTTTTTTTAGGT	*mir-41* qPCR
KK188: mir-42fqPCR	GTCACCGGGTTAACATCT	*mir-42* qPCR
KK189: mir-42rqPCR	GTCCAGTTTTTTTTTTTTTTTCTGT	*mir-42* qPCR
KK190: mir-86fqPCRref	AGTAAGTGAATGCTTTGCCA	*mir-86* qPCR
KK191: mir-86rqPCRref	GTCCAGTTTTTTTTTTTTTTTGACT	*mir-86* qPCR
KK192: mir-248fqPCRref	GATACACGTGCACGGA	*mir-248* qPCR
KK193: mir-248rqPCRref	CAGTTTTTTTTTTTTTTTGAGCGT	*mir-248* qPCR
KK194: mir-794fqPCRref	CAGTGAGGTAATCATCGTTGT	*mir-794* qPCR
KK195: mir-794rqPCRref	GTCCAGTTTTTTTTTTTTTTTAGTGA	*mir-794* qPCR
KK196: mir-35fNheI	GCTGCTAGCTCTCGGATCAGATCGAGCC	mir-35-51 promoter region amplification
KK197: mir-35RNcoI	CGACCATGGGGAAAAGATCGAGCCACTGC	mir-35-51 promoter region amplification
KK198: mir-35fBamHI	GCTGGATCCTCTCGGATCAGATCGAGCC	mir-35-51 promoter region amplification
KK199: mir-35SacI	CGAGAGCTCGGAAAAGATCGAGCCACTGC	mir-35-51 promoter region amplification
KK238 Promsup-26FhindIII	GGGAAGCTTTTAGCTAGCTCGCAGAGCC	*sup-26* promoter region amplification
KK239 Promsup-26RbamHI	GGGTGGATCCCTTGAATTATTATGATGATG	*sup-26* promoter region amplification
KK240 Prommir-35FhindIII	GGGAAGCTTATCCGTCCACGTCTTCAATC	*mir-35-51* promoter region amplification
KK241 Prommir-35RbamHI	GGGTGGATCCATAATAGTTGGGAATGG	*mir-35-51* promoter region amplification
KK243: sup-26-3’UTRfEcoRI	GGGCgaattcATGGACAGGACAACGTC	*sup-26* 3'UTR umplification
KK244: sup-26-3’UTRrApaI	GGTCgggcccCTCTGGAATCATTTATTTAC	*sup-26* 3'UTR umplification
KK281: sup-26-RNAiF	GTTTTCCCAGTCACGACGTT	sup-26 RNAi
KK282: sup-26-RNAiR	TGGATAACCGTATTACCGCC	sup-26 RNAi
mir-35-41 specific rt primer	TCTCGGATCAGATCGAGCCA	Pri-mir-35-41
Pri/pre mir-38 F	GTGAGCCAGGTCCTGTTC	Pri/pre-mir-38
Pri/pre mir-38 R	TGAGTCACAGGTCCTACTC	Pri/pre-mir-38

Supplementary References

1. Brenner, S. (1974). The genetics of Caenorhabditis elegans. Genetics *77*, 71-94.
2. Mello, C.C., Kramer, J.M., Stinchcomb, D., and Ambros, V. (1991). Efficient gene transfer in C.elegans: extrachromosomal maintenance and integration of transforming sequences. Embo J *10*, 3959-3970.
3. Mapes, J., Chen, J.T., Yu, J.S., and Xue, D. (2010). Somatic sex determination in Caenorhabditis elegans is modulated by SUP-26 repression of tra-2 translation. P Natl Acad Sci USA *107*, 18022-18027.
4. Chomczynski, P., and Sacchi, N. (1987). Single-step method of RNA isolation by acid guanidinium thiocyanate-phenol-chloroform extraction. Anal Biochem *162*, 156-159.
5. Vandesompele, J., De Preter, K., Pattyn, F., Poppe, B., Van Roy, N., De Paepe, A., and Speleman, F. (2002). Accurate normalization of real-time quantitative RT-PCR data by geometric averaging of multiple internal control genes. Genome Biol *3*, RESEARCH0034.
6. Schmittgen, T.D., Lee, E.J., Jiang, J., Sarkar, A., Yang, L., Elton, T.S., and Chen, C. (2008). Real-time PCR quantification of precursor and mature microRNA. Methods *44*, 31-38.
